# Intronic cleavage and polyadenylation regulates gene expression during DNA damage response through U1 snRNA

**DOI:** 10.1038/celldisc.2016.13

**Published:** 2016-06-14

**Authors:** Emral Devany, Ji Yeon Park, Michael R Murphy, George Zakusilo, Jorge Baquero, Xiaokan Zhang, Mainul Hoque, Bin Tian, Frida E Kleiman

**Affiliations:** 1 Chemistry Department, Belfer Research Building, Hunter College and Graduate Center, City University of New York, New York, NY, USA; 2 Department of Microbiology, Biochemistry and Molecular Genetics, Rutgers New Jersey Medical School, Newark, NJ, USA

**Keywords:** alternative polyadenylation, DNA damage response, U1 snRNP

## Abstract

The DNA damage response involves coordinated control of gene expression and DNA repair. Using deep sequencing, we found widespread changes of alternative cleavage and polyadenylation site usage on ultraviolet-treatment in mammalian cells. Alternative cleavage and polyadenylation regulation in the 3ʹ untranslated region is substantial, leading to both shortening and lengthening of 3ʹ untranslated regions of genes. Interestingly, a strong activation of intronic alternative cleavage and polyadenylation sites is detected, resulting in widespread expression of truncated transcripts. Intronic alternative cleavage and polyadenylation events are biased to the 5ʹ end of genes and affect gene groups with important functions in DNA damage response and cancer. Moreover, intronic alternative cleavage and polyadenylation site activation during DNA damage response correlates with a decrease in U1 snRNA levels, and is reversible by U1 snRNA overexpression. Importantly, U1 snRNA overexpression mitigates ultraviolet-induced apoptosis. Together, these data reveal a significant gene regulatory scheme in DNA damage response where U1 snRNA impacts gene expression via the U1-alternative cleavage and polyadenylation axis.

## Introduction

Almost all eukaryotic mRNA precursors undergo a co-transcriptional modification at the 3ʹ end, which includes two coupled steps, cleavage and polyadenylation [1, 2]. Cleavage/polyadenylation (C/P) involves recognition of upstream and downstream cis elements around the C/P site (known as pA) by the C/P complex [3, 4]. While a relatively simple signal sequence in the precursor mRNA is required for the reaction, many interactions between a large number of protein factors are necessary for the correct formation of the C/P complex [3]. In addition to factors in the core C/P complex, it has been shown that splicing factors can play roles in 3ʹ end processing. U1 snRNP (or U1) has been implicated in inhibition of C/P via poly(A) polymerase [5–7]. This mechanism has recently been suggested to play a key role in controlling transcript length [8, 9]. In addition, U2 snRNP factors have been shown to interact with core C/P factors [10].

Well over half of the mammalian genes contain more than one pA, leading to expression of alternative cleavage and polyadenylation (APA) isoforms [11]. APA is highly dynamic across tissue types [12, 13], in cell proliferation and differentiation [14, 15], and in response to extracellular cues [16]. Most APA sites are located in the 3ʹ untranslated region (3ʹUTR) of mRNA [17]. As 3ʹUTRs contain various cis elements for post-transcriptional control, such as microRNA target sites and AU-rich elements, 3ʹUTR-APA can significantly impact mRNA metabolism. In addition, a sizable fraction of genes harbor pAs in introns [17]. Intron-APA can result in change of coding sequences of mRNA, impacting the proteome. The core mammalian C/P machinery and additional cis elements around the pA are responsible for the selection among APA sites [18–20]. Interestingly, in keeping with U1’s role in C/P, recent studies have shown that inhibition of U1 function leads to activation of intron-APA events, resulting in shorter transcripts [8, 21].

The DNA damage response (DDR) occurs on a number of environmental exposures, such as ultraviolet (UV) irradiation, and involves functional and structural changes in a number of nuclear proteins, resulting in a coordinated control of gene expression and DNA repair. One key aspect of the response is the transient decrease of the cellular levels of mRNA following UV irradiation and its recovery [22, 23]. Although the mechanisms involved in this response are still not completely resolved, it has been determined that the UV-induced inhibition of both transcription [24] and mRNA 3ʹ processing [25] are responsible for the decrease in mRNA levels. Both 3ʹ end formation and transcription are affected in a similar time frame after DNA damage, resulting in a general, transient decrease of the cellular levels of polyadenylated transcripts [25]. mRNA levels of genes involved in DDR appear to be specifically regulated at the 3ʹ-end processing step [26]. Tumor suppressors and DNA repair factors whose expression is commonly compromised in most cancers, such as BARD1 and p53, have functional interactions with the 3ʹ end processing factor CstF-50 and PARN deadenylase, resulting in the regulation of mRNA 3ʹ processing during DDR [25, 27–33]. In addition, we have found that PARN deadenylase has a role in decreasing the levels of short-lived mRNAs involved in the regulation of cell growth, differentiation and DDR, and keeping their expression levels low under non-stress conditions [30, 33]. The existence of redundant mechanisms to control mRNA steady-state levels during DDR highlights the importance of the transcription/RNA processing machineries in this response.

Here we explore the mechanisms and consequences of APA on UV-induced DNA damage. Using 3ʹ region extraction and deep sequencing (3ʹREADS), we show widespread changes of intron-APA and 3ʹUTR-APA on UV treatment in mammalian cells. Distinct APA changes at different time points after UV treatment affect many genes involved in DDR and cancer. Intron-APA upregulation correlates with a decrease in U1 snRNA levels after UV-induced DNA damage. Importantly, overexpression of U1 snRNA reverses UV-induced intron-APA and mitigates the apoptosis caused by UV. Our results indicate that the U1–APA axis is an important part of gene regulatory mechanism in DDR.

## Results

### Analysis of UV-induced APA by 3ʹREADS

**Figure 1:**
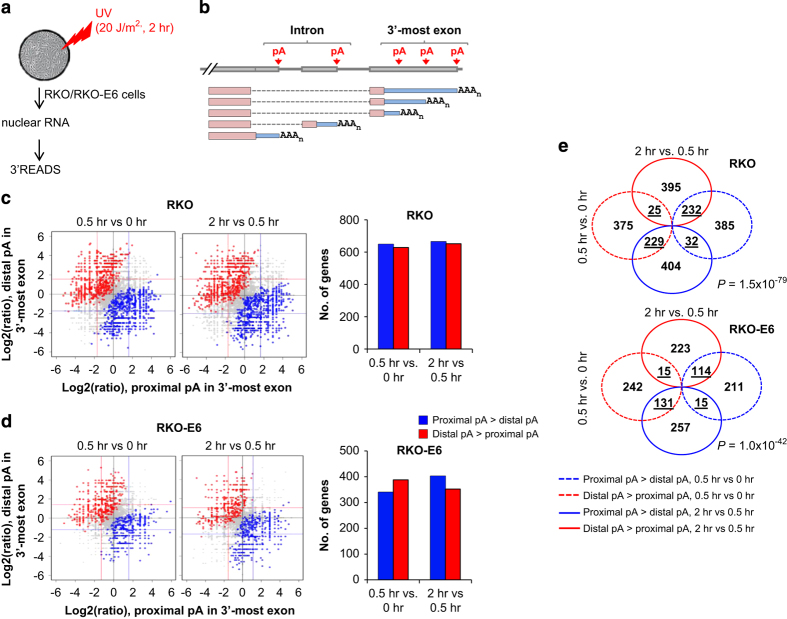
Regulation of 3ʹUTR-APA in both RKO and RKO-E6 cells after UV treatment**.** (**a**) Experimental design for analysis of APA in UV-treated cells using 3ʹREADS. (**b**) Schematic showing two types of APA examined in this study, namely intron-APA and 3ʹUTR-APA. (**c**, **d**) Comparison of 3ʹUTR-APA in RKO and RKO-E6 cells. Top 2 most abundant 3ʹUTR isoforms were chosen for each gene. Log2 ratios of expression are plotted between two isoforms. APA changes were examined in two time windows (0.5 vs 0 h, and 2 vs 0.5 h) and considered significant when *P*-value<0.05 (Fisher’s exact test). Blue dots represent genes with upregulated proximal pA isoform (3ʹUTR shortened), and red dots represents genes with upregulated distal pA isoform (3ʹUTR lengthened). Grey dots are genes that have no significant regulation. Colored lines indicate median values for blue or red dots. Number of genes with regulated 3ʹUTR is shown on the right plot. Blue and red dots on the left correspond to genes in blue and red bars, respectively. (**e**) Venn diagram comparing significantly regulated 3ʹUTR-pA isoforms for both RKO and RKO-E6 cells. *P*-value (Fisher’s exact test) indicates bias of distribution of the numbers in four overlapping areas (underlined). APA, alternative cleavage and polyadenylation; 3′READS, 3′ region extraction and deep sequencing; UTR, untranslated region; UV, ultraviolet.

Previous studies indicated that 3ʹ end processing is regulated during DDR [25, 27–33]. To examine how APA is modulated in DDR, we treated colon carcinoma RKO cells with UV irradiation, followed by recovery for either 0.5 or 2 h. To determine whether the effect of UV treatment on APA was general or specific to certain genetic background, we included in our study RKO-E6 (low p53 levels) cell line, which is isogenic to the RKO cell line. To mitigate the effect of differential regulation of APA isoforms through mRNA decay in cytoplasm and in keeping with our previous work to study functional effect of DNA damage using nuclear RNA and factors [30–34], nuclear RNA was extracted and subjected to 3ʹREADS (Figure 1a), a recently developed deep sequencing method for analysis of APA isoform expression genome wide [17]. We examined relative expression of APA isoforms that used pAs in the 3ʹ-most exon, which typically have different 3ʹUTRs, as well as intronic pAs, which have different coding sequences and 3ʹUTRs (Illustrated in Figure 1b).

To simplify the analysis of APA in 3ʹ-most exons, where a variable number of pAs can exist [11], we selected top two 3ʹUTR-APA isoforms for each gene with the most number of reads and examined their relative expression. For RKO cells, we identified 1 278 and 1 317 genes that displayed isoform expression changes in the 0–0.5 h and 0.5–2 h time windows, respectively (Fisher exact test, *P*<0.05; Figure 1c). For RKO-E6, we identified 728 and 755 genes with significant 3ʹUTR-APA changes in the 0–0.5 h and 0.5–2 h time windows, respectively (Figure 1d). The fact that fewer genes underwent 3ʹUTR-APA in RKO-E6 cells than in RKO cells suggests a potential role of p53 in impacting the extent of 3ʹUTR-APA regulation during DDR. Overall, for both cell lines, the number of genes which had upregulated distal pA isoform was similar to that of genes which had upregulated proximal pA isoform in both windows, indicating that there was no global direction for 3ʹUTR length changes under these conditions. It is noteworthy that in both time windows and for both cells lines, upregulation of proximal pA isoforms was accompanied with a similar magnitude downregulation of distal pA isoforms and vice versa (similar *x*-axis and *y*-axis median values for blue and red dots in Figure 1c and d), indicating that APA isoform expression regulation was generally due to changes of pA choice rather than differences in isoform stability (which would cause different magnitudes of regulation).

Interestingly, the APA pattern in the 0–0.5 h window was largely different than the 0.5–2 h window for both cell lines (Figure 1e); a group of pAs in fact had an opposite regulatory trend between the two time windows. This result indicates widespread and dynamic 3ʹUTR-APA regulation during the progression of DDR. Consistently, Gene Ontology (GO) analysis indicated that different biological processes were affected by 3ʹUTR-APA in the two windows (Supplementary Table S1). For example, APA regulation was significantly enriched for genes associated with ‘cell redox homeostasis’, ‘cellular homeostasis’ and ‘regulation of cellular component organization’ in the 0.5–2.0 h window, whereas ‘protein localization to endoplasmic reticulum’, ‘negative regulation of transport’ and ‘membrane protein proteolysis’ were found to be associated with genes with APA regulation in the 0–0.5 h window. Three example genes are shown in Supplementary Figure S1.

**Figure 2:**
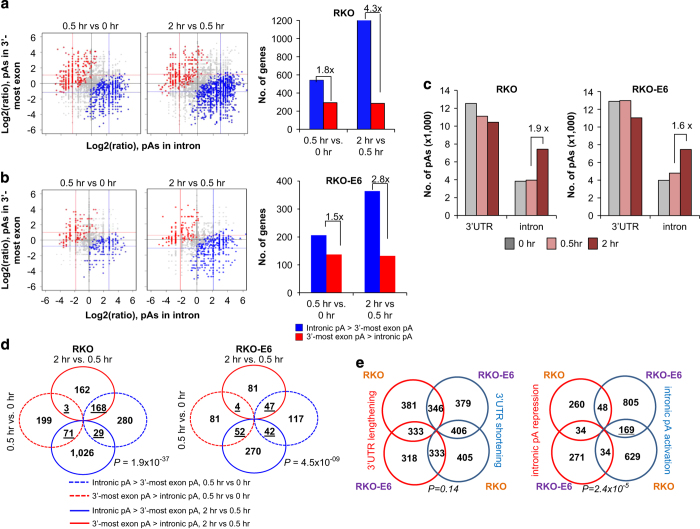
Regulation of intron-APA in both RKO and RKO-E6 cells after UV treatment. (**a**, **b**) Comparison of expression between intronic pA isoforms and all isoforms using pAs in the 3ʹ-most exon in RKO and RKO-E6 cells. Blue dots correspond to genes with upregulated proximal pA isoform (intronic pA activation) and red dots correspond to genes with upregulated distal pA isoform (intronic pA repression). Grey dots are genes that have no significant regulation. Colored lines indicate median values for blue or red dots. Number of genes with significantly regulated intron-APA events is shown on the right. Ratio of number of genes in the blue bar to that of genes in the red bar is indicated. (**c**) Distribution of pAs detected in 3ʹUTRs or introns in samples from both RKO and RKO-E6 cells. Only pAs with read number greater than 5% of all reads of the gene were used. (**d**) Venn diagram comparing genes with significantly regulated intronic pA isoforms in two windows in samples from both RKO and RKO-E6 cells. (**e**) Venn diagram comparing significantly regulated APA events in RKO and RKO-E6 cells. Significance was determined by RED (relative expression difference), with the cutoff of >1 or <−1. (left) 3ʹUTR-APA events. (right) intron-APA events. Statistical analysis of overlapping genes was performed by the Fisher’s exact test. APA, alternative cleavage and polyadenylation; 3′READS, 3′ region extraction and deep sequencing; UTR, untranslated region; UV, ultraviolet.

A large fraction of human pAs are located in introns [35]. We next compared expression of isoforms using intronic pAs with those using 3ʹ-most exon pAs for RKO and RKO-E6 cells. As with 3ʹUTR-APA events, fewer genes underwent intron-APA in RKO-E6 cells than in RKO cells, suggesting a role of p53 in the regulation (Figure 2a and b). Much to our surprise, intronic pA isoforms were greatly upregulated compared with 3ʹ-most exon pA isoforms in both cell lines. This trend was much more conspicuous in the 0.5–2 h window than the 0–0.5 h window, in which 4.3- and 1.8-fold more genes had upregulated intronic pA isoforms than had upregulated 3ʹ-most exon pA isoforms, respectively (Figure 2a). A similar trend was observed for RKO-E6 cells albeit to a lesser extent (2.8- and 1.5-fold, Figure 2b). In contrast to 3ʹUTR-APA regulation, the magnitude of upregulation of intronic pA isoforms in both cell lines was greater than that of downregulation of 3ʹ-most exon pA isoforms (different *x*-axis and *y*-axis median values for blue and red dots in Figure 2a and b), in line with the fact that intronic pAs isoforms are typically expressed at much lower levels than 3ʹUTR pA isoforms. Consistently, greater numbers of intronic pAs were detected in 2 h samples than in 0 or 0.5 h samples by 1.9- and 1.6-fold for RKO and RKO-E6, respectively (Figure 2c). Six example genes are shown in Supplementary Figure S2. Together, these results suggest that while p53 expression may impact the extent of APA regulation, it does not affect the direction of regulation.

Similar to the 3ʹUTR-APA result, genes with regulated intronic pAs in the two time windows analyzed are largely different (Figure 2d). GO analysis (Supplementary Table S2) indicated that upregulated intronic pAs in the 0–0.5 h window were enriched for genes associated with several GO terms, such as ‘regulation of protein kinase B signaling cascade’ and ‘modification of morphology or physiology of other organism involved in symbiotic interaction’, and those in the 0.5–2 h window were enriched for genes associated with ‘regulation of transcription from RNA polymerase II promoter’, ‘response to DNA damage stimulus’, ‘nucleocytoplasmic transport’ and so on. A significant number of regulated intronic APA events overlapped between RKO and RKO-E6 cells (*P*=2.4×10^−5^, Fisher’s exact test, Figure 2e, right panel), suggesting that the effect of UV treatment on intronic APA is not cell type specific. Notably, the extent of overlap is greater than that for 3ʹUTR-APA (*P*=0.14, Fisher’s exact test, Figure 2e, left panel), suggesting that intron-APA is less regulated by p53 than is 3ʹUTR-APA.

**Figure 3:**
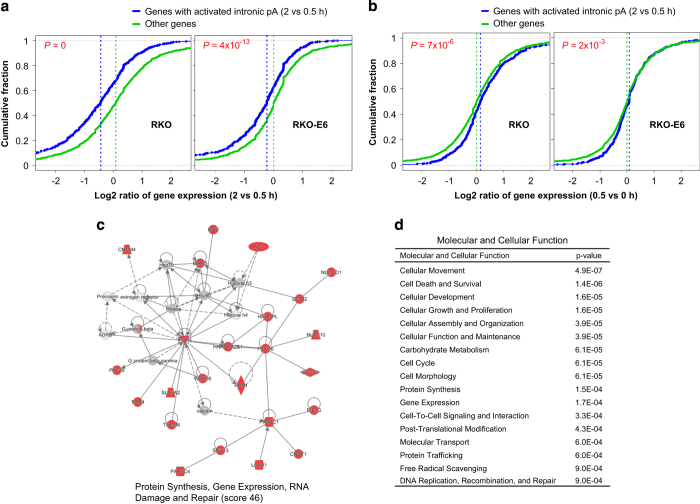
Relationship between intronic APA and gene expression regulation**.** (**a**) Gene expression changes vs intron-APA regulation. The blue line is for genes with activated intronic pA in 2 vs 0.5 h (RED<−1), and green line for other genes. *X*-axis indicates log2 ratio of gene expression in 2 vs 0.5 h in RKO (left) and RKO-E6 (right). *Y*-axis is cumulative fraction. The median value of each group is indicated by a dotted vertical line. The *P*-value is based on comparison of two groups using the Kolmogorov–Smirnov test. (**b**) As in **a** except that gene expression changes are based on 0.5 vs 0 h. Note that intronic regulation is based on 2 vs 0.5 h (RED<−1) as in **a**. (**c**, **d**) Significant functional categories enriched with genes with significant intronic APA regulation in both RKO and RKO-E6 cells (underlined in the Venn diagram in Figure 2e, right panel), as analyzed by the Ingenuity Pathway Analysis Network (**c**) and Molecular and Cellular Function terms (**d**). In the network, red nodes indicate genes with significant intronic APA regulation. APA, alternative cleavage and polyadenylation.

We then asked how intron-APA regulation was related to gene expression (Figure 3a and b, Supplementary Figure S6). Our data indicate that genes with intronic pA activation between 0.5 and 2 h were more likely to be downregulated in the same period, as compared with other genes (Figure 3a), suggesting that intronic APA can inhibit gene expression by generating truncated transcripts. Interestingly, we also found that the same genes with intronic pA activation between 0.5 and 2 h were also more likely to be upregulated between 0 and 0.5 h after UV treatment (Figure 3b), suggesting that intronic APA might serve as a mechanism to regulate gene expression of factors involved in the response, such as POLR2A and cyclin-dependent kinase inhibitor 1A (CDKN1A), assuring that cells react to damage response in a controlled and timely manner. Consistent with this and the results shown in Supplementary Table S2, Ingenuity Pathway Analysis showed that genes with significant intronic APA regulation in both RKO and RKO-E6 cells were associated with pathways highly relevant to DDR (Figure 3c and d).

### Validation of UV-induced APAs detected by 3ʹ READS

**Figure 4:**
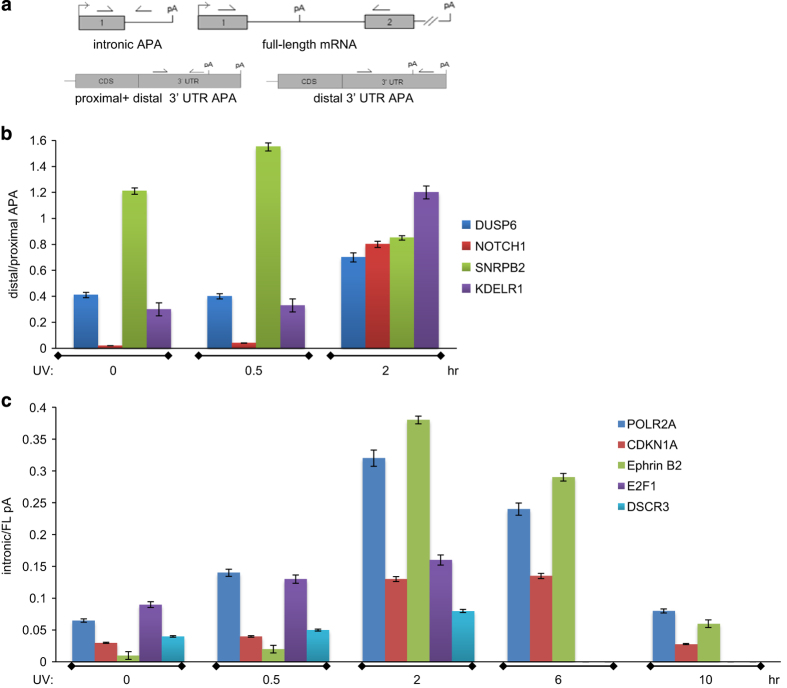
Effect of UV treatment on APA. (**a**) Schematic representation of amplified regions to detect 3ʹUTR and intronic APA. HCT116 were treated with UV irradiation and allowed to recover for indicated times, and then harvested. cDNA was prepared using oligo(dT) primers from nuclear RNA and used for PCR and qPCR reactions with primers specific for intronic APA, full-length mRNA, and 3ʹUTR (proximal and distal) APA. (**b**) qRT–PCR analysis of HCT116 samples for the effect of UV on 3ʹUTR pA choice. The ratio of distal/proximal of each analyzed gene is shown. Samples were prepared as described in **a**. The qRT–PCR values were calculated from three independent biological samples by triplicate. (**c**) Intron-APA is transiently upregulated on UV-induced DNA damage. Samples were analyzed as in **a** but the ratio of intronic/full-length of each mRNA is shown. The qRT–PCR values were calculated from three independent samples. APA, alternative cleavage and polyadenylation; cDNA, complementary DNA; qRT–PCR, quantitative reverse transcription–PCR; UV, ultraviolet.

To validate our genome-wide analysis, we examined 3ʹUTR- and intron-APAs for genes following the strategies shown in Figure 4a. To further confirm that the effect of UV treatment on intronic APA is not a cell type-specific effect, we extended our study to colon carcinoma HCT116 cells. Briefly, after recovery from UV treatment, nuclear RNA was isolated from colon carcinoma HCT116 and RKO cells and complementary DNA (cDNA) was synthesized by reverse transcription using oligo(dT) primers. Quantitative reverse transcription–PCR (qRT–PCR) was performed with these cDNA as template. HCT116 results are shown in Figure 4b and c. Three primers were used to detect intron-APA products (short isoform) and full-length mRNAs (long isoform): the forward primers were located in the upstream exons of regulated intronic pAs of studied genes and the two reverse primers corresponded to either the intron containing the pA (for detection of short isoform) or the downstream exon (for detection of long isoform). A similar strategy was used to detect 3ʹUTR-APA products: a common forward primer in the 3ʹUTR and the two reverse primers corresponded to either upstream (for detection of total 3ʹUTR-APA) or downstream (for detection of distal-APA isoforms) from the used pA. The values for the proximal APA were calculated by subtracting the distal-APA values from the total 3ʹUTR-APA values.

As shown in Figure 4b, our analysis for 3ʹUTR-APAs included genes involved in different biological pathways (Supplementary Table S1) with functions in DDR and cancer, such as small nuclear ribonucleoprotein polypeptide B (SNRPB2) [36, 37], endoplasmic reticulum protein retention receptor 1 (KDELR1) [38, 39]; Notch homolog 1 translocation-associated (NOTCH1) [40, 41] and dual specificity phosphatase 6 (DUSP6) [42, 43]. Consistent with the 3ʹREADS results (Figure 1c), the analysis of UV-induced 3ʹUTR-APA in the 0–0.5 h window indicated that each individual gene did not show a major change in the distal/proximal ratio. However, UV treatment in the 0–2 h window induced changes in the usage of pA for individual genes, favoring either distal (KDELR1, NOTCH1 and DUSP6) or proximal (SNRPB2) pAs. The analysis shown in Figure 1c might represent the overall behavior of the total genes analyzed, indicating that there was no global direction of 3ʹUTR length changes under these conditions.

Our analysis for intron-APAs included genes with important functions in DDR and cancer (Figure 4c), namely cyclin-dependent kinase inhibitor 1A (CDKN1A, p21) [44, 45], polymerase (RNA) II (DNA directed) polypeptide A (POLR2A, RNA polymerase II) [46, 47], Ephrin B2 (EFNB2) [48, 49], E2F transcription factor 1 (E2F1) [50, 51] and Down syndrome critical region gene 3 (DSCR3) [52, 53]. Importantly, based on IPA analysis, CDKN1A and POLR2A were at the hub of the networks significantly associated with intronic pA activation in the 0.5–2 h window (Supplementary Figure S3A and B). Consistent with the 3ʹREADS analysis results, UV treatment induced the formation of intron-APA transcripts that were polyadenylated (Figure 4b and c). Interestingly, the increase in intron-APA isoforms was observed from 2 to 6 h after UV treatment, but these shorter isoforms decrease after 10 h, reaching the levels of untreated cells (Figure 4c). Similar results were observed with RKO cells (not shown), suggesting that UV-mediated regulation of intronic APA is not a cell type-specific effect. The transient nature of these intron-APA isoforms is consistent with previously characterized responses to DNA damage [25, 30]. The sequences of the second intron was not detected in any of the mRNAs samples analyzed (Supplementary Figure S4A), indicating that intron-APA was induced within the first intron of the target mRNAs. Together, these results indicate the UV treatment induce the usage of intronic pAs of genes involved in DDR, suggesting a possible role for these intron-APA events in controlling gene expression during the response.

### Features of UV-induced intronic APA events

**Figure 5:**
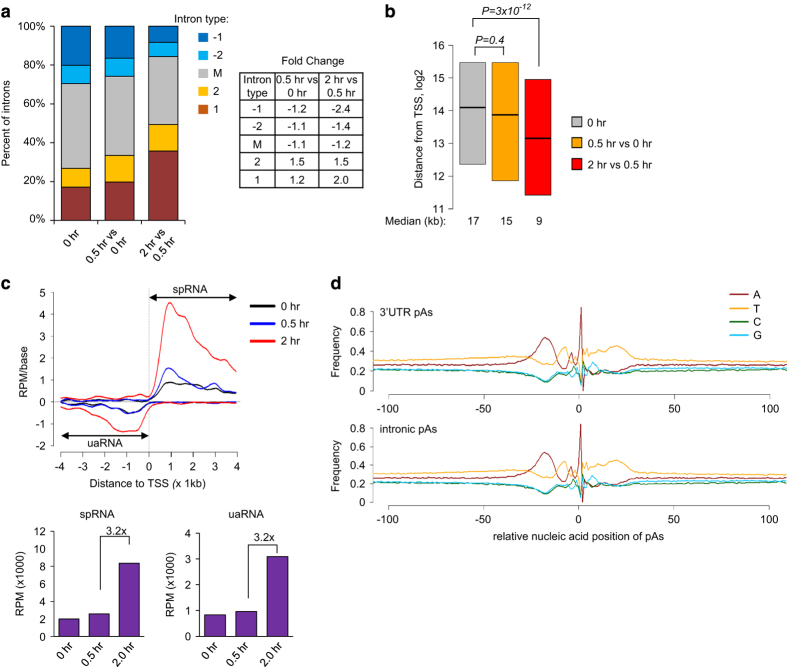
Regulation of intronic APA and C/P events around transcription start site (TSS) in response to UV**.** (**a**) Distribution of introns with activated pAs in genes. 1, first intron; 2, second intron; M, middle introns; −2, second to the last intron; −1, last intron. Single introns were excluded from this analysis. The number of introns falling into multiple types, for example, both +1 and −1 types, was evenly divided in calculation. Introns with pAs at 0 h were used as control. Changes of percent of introns with activated pAs at 0.5 vs 0 h or 2 vs 0.5 h are shown in a table next to the plot. (**b**) Boxplot showing distance from the transcription start site (TSS) to intronic pAs. Only the 25th to 75th percentile values are shown. The 0 h data are based on all detected intronic pAs, and the 0.5 vs 0 h and 2 vs 0.5 h data are based on activated intronic pAs. *P*-values were based on Wilcoxon test comparing the 0.5 vs 0 h or 2 vs 0.5 h values with those of control (0 h). (**c**) Distribution of pAs around the TSS. Top panel: all detected pAs in three samples are plotted, and are shown as reads per million (RPM) per base. The pAs within 4 kb from the TSS on the anti-sense strand are called upstream anti-sense RNA (uaRNA) pAs, and those within 4 kb from the TSS on the sense strand are called sense proximal RNA (spRNA) pAs. For uaRNA pAs, we discarded pAs that were associated with any known genes, and for spRNA pAs we excluded pAs that were located in the 3ʹ-most exon of genes. Bottom panel: the amounts of uaRNAs or spRNAs are shown in bar graphs. The ratio of transcript amount in the 2 h sample to that in the 0.5 h sample for spRNA pAs (top) or uaRNA pAs (bottom) is indicated. (**d**) Distribution of nucleotides as a function of base position around the pA identified by 3ʹREADS. The upstream and downstream regions around the pA were analyzed, spanning from −100 nt to 100 nt, with the pA set at 0 nt. The *y*-axis indicates frequency of each type of nucleotide at a given position. Top panel: nucleotide frequency around 3ʹUTR pAs (14 058 in total). Bottom panel: nucleotide frequency of intronic pAs (6 750 in total). APA, alternative cleavage and polyadenylation; 3′READS, 3′ region extraction and deep sequencing.

We next examined features of introns that harbor activated pAs in DDR in our 3ʹREADS data. Strikingly, we found the activated intronic pAs are highly enriched (>1.5-fold above background) in 5ʹ introns, with the most notable enrichment being pAs in the first intron in the 0.5–2 h window (2-fold above background) (Figure 5a). Consistently, we found that the distance from the activated pAs in the 0.5–2 h window to transcription start site (TSS) was significantly shorter than those in control cells (*P*=3×10^−12^) (Figure 5b), a trend not seen for activated pAs in the 0–0.5 h window (*P*=0.4). Further analysis of intron features indicated that introns harboring activated pAs are larger and had stronger 5ʹ splice site (5ʹSS) compared with other introns (Supplementary Figure S5). However, these distinct features were not significantly different from other first introns. Note that the upregulated intronic pAs of POLR2A and EFNB2 are both located in the first intron, and the APA event of CDKN1A was either in the first intron or a 5ʹ intron depending on the TSS (Supplementary Figure S2). Interestingly, in addition to the sense strand pAs, we also noticed in the 0.5–2 h window a general upregulation of transcripts using pAs within 4 kb from the TSS on the anti-sense strand (Figure 5c). These transcripts were previously named upstream anti-sense RNA (uaRNA) or PROMPTs [54, 55]. Their activation in the 0.5–2 h window suggests a unique mechanism regulating RNAP II activity around the TSS in this phase of DDR response. We also determined the distribution of nucleotides around the 3ʹUTR and intronic pAs identified by 3ʹREADS (Figure 5d). The base composition profiles upstream and downstream from the pA for these two groups of pAs were highly similar and consistent with those of known pAs [11], indicating that the 3ʹUTR and intronic pAs detected by 3ʹREADS were genuine pAs with similar surrounding cis elements.

### Role of U1 RNA levels in intronic APA events during DDR

**Figure 6:**
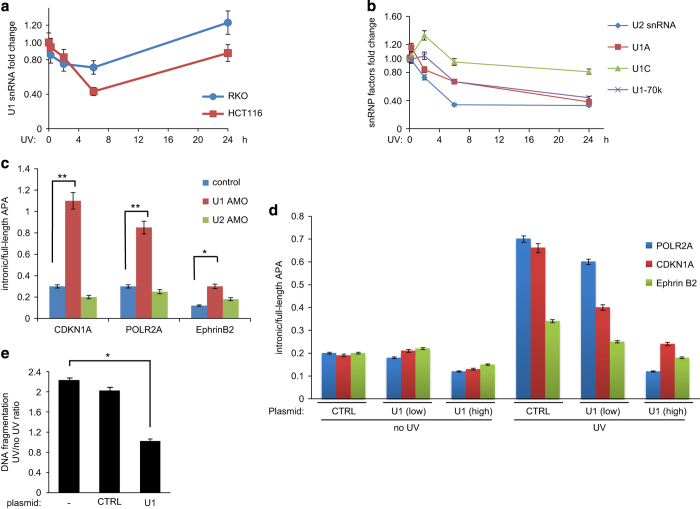
U1 RNA levels inversely correlate with intronic APA. (**a**) U1 snRNA levels decrease after UV treatment. HCT116 and RKO cells were treated with UV irradiation and allowed to recover for the indicated times, and then harvested. Nuclear RNA was isolated and cDNA was prepared using random primers. qRT–PCR reaction was performed with primers specific for U1 snRNA. qRT–PCR products of actin were used as endogenous control. The values from each sample were normalized to non-treated samples. The qRT–PCR values were calculated from three biological samples by triplicate in each determination. (**b**) Changes in the levels of spliceosome complex components on UV treatment. HCT116 cells were treated with UV irradiation and analyzed as in Figure 4a by qRT–PCR reaction with primers specific for U2 snRNA, U1A, U1C and U1-70K. The qRT–PCR values were calculated from three independent samples. (**c**) Functional depletion of U1 snRNA, but not U2 snRNA, causes an increase in intronic pA. HCT116 cells were transfected with control or anti-sense morpholino targeting U1 snRNA (U1 AMO) or U2 snRNA (U2 AMO). cDNA was prepared and used in qRT–PCR assays as in Figure 4c. The qRT–PCR values were calculated from three biological samples were analyzed by triplicate in each determination. (**d**) Overexpression of U1 RNA abolishes UV-induced intronic APA. HCT116 cells were transfected with two concentrations of either control or U1 snRNA expressing vectors and treated with UV irradiation. Nuclear RNA was used to prepare cDNA, which was used in qRT–PCR reactions as in Figure 2b. The qRT–PCR values were calculated from three independent samples. (**e**) Overexpression of U1 RNA decreases the levels of UV-induced apoptosis in HCT116 cells. The DNA fragmentation was calculated from three independent samples. APA, alternative cleavage and polyadenylation; cDNA, complementary DNA; qRT–PCR, quantitative reverse transcription–PCR; UV, ultraviolet.

The activation of promoter-proximal pAs in 2 vs 0.5 h is reminiscent of APA regulation by U1 snRNP: functional depletion of U1 RNA shortens mRNAs due to usage of promoter-proximal cleavage and polyadenylation signals [9]. Earlier studies have shown a decrease in U1 and U2 small RNA levels in HeLa cells on UV treatment [56]. Therefore, we examined whether intronic APA was triggered by U1 RNA reduction in response to UV irradiation. First, we detected the effect of UV treatment on the levels of U1 RNA by qRT–PCR using HCT116 and RKO cells (Figure 6a). Cells were treated with UV irradiation and allowed to recover for the indicated time points. Consistent with the studies of Eliceiri and Smith [56], our qRT–PCR analysis of nuclear RNA samples from these cells showed a transient decrease in U1 RNA levels on UV treatment (Figure 6a). Although a decrease in U1 RNA was detected as early as 0.5 h after UV treatment, the lowest level of U1 RNA was observed 6 h after UV treatment for both cell lines. The levels of U1 RNA increased 24 h after UV treatment, reaching the levels of untreated cells. Extending those studies, we analyzed the levels of other components of the U1 and U2 snRNPs by qRT–PCR. After UV treatment, a decrease was also observed in U2 RNA, U1A and U1-70K mRNAs of U1 snRNP (Figure 6b). No significant changes were observed in U1C levels. Thus, among all the molecules examined, U1 snRNA levels (Figure 6a) correlated the best with the changes in intronic/full-length APA levels (Figure 4c). Previous studies indicate that U1 snRNPs, such as U1A and U1-70K, are inhibitors of 3ʹ end processing and intronic APA (reviewed in 57). Our studies indicate that the decrease of these U1 snRNPs early in DDR might increase intronic APA. However, only U1 snRNA levels correlate with the decrease in intronic APA later in the response, suggesting that other components of U1 snRNP might not be at the rate-limiting level during DDR.

Importantly, functional depletion of U1 RNA using morpholino oligonucleotides increased significantly the ratio of intron/full-length APA isoforms for POLR2A, CDKN1A and EFNB2 (Figure 6c). Strikingly, using low concentrations of U1 snRNA AMO, the changes in the intronic/full-length ratio by U1 RNA depletion were similar in magnitude to that observed after UV treatment (compare Figures 4b and 6c). As previously described [9], we did not detect sequences of the second intron in any of the mRNA samples analyzed at low concentrations of U1 snRNA AMO (Supplementary Figure S4B), indicating that the moderate functional decrease in U1 levels was insufficient to inhibit splicing. However, at higher concentrations, second intron inclusion was observed for the genes analyzed (Supplementary Figure S4B). U2 RNA depletion using morpholino did not increase the usage of examined intronic pAs (Figure 6c). This is consistent with previous studies showing that decrease of U2 snRNP levels has a different impact on intronic APA [21]. Supporting these results, overexpression of U1 snRNA reverses the UV-induced increase of intron-APA (Figure 6d) and apoptosis (Figure 6e). Together, these results indicate that the UV-induced downregulation of U1 snRNA, not other components of the U1 snRNP, is chiefly responsible for the activation of intronic APA sites during DDR.

## Discussion

Here we report significant activation of intronic pAs on UV treatment in mammalian cells, adding a new layer of gene regulation in the cellular response to UV-induced DNA damage. At the center of this mechanism is U1 snRNA, one of the component of U1 snRNP, which has previously been shown to play an important role not only in splicing but also in 3ʹ end processing [5, 20]. Our data for the first time provides a cellular response/pathway that is affected by the U1–APA axis and shows that downregulation of the U1 snRNA level is the controlling step for intronic APA in DDR. This mechanism results in activation of pAs located near TSS, mostly in the first intron, and serves as a rapid (within 2 h after UV) strategy to regulate expression of affected genes. Given the different correlations between intronic pA activation and gene expression changes in 0.5 vs 2 h and 0 vs 0.5 h windows, it is plausible that this mechanism assures that the expression of factors involved in the response, such POLR2A and CDKN1A, occurs in a controlled and timely manner. Consistent with this, genes with significant intronic APA regulation belong to pathways activated during DDR and downregulation of gene expression was more likely to be associated with intronic APA.

Our results indicate that similar changes in APA events occur in different cell lines in a similar timeframe, suggesting that UV-mediated regulation of intronic APA and 3ʹUTR-APA are not a cell type-specific effect. However, our results also indicate that p53 expression may impact the extent of APA regulation during DDR. This is not surprising given that p53 is involved in different aspects of gene expression regulation during DDR, such as controlling transcription, mRNA stability and/or translation [58, 59]. Further experiments are needed to reveal the exact role of p53 in APA regulation in DDR.

Although transcriptional and protein level changes of POLR2A and CDKN1A are known mechanisms of UV response in mammalian cells, the UV-induced regulation of their APA isoforms and steady-state levels of their transcripts have never been reported. A fraction of the largest subunit of POLR2A decreases by DNA damage-induced ubiquitination followed by proteasomal degradation in mammalian cells [60, 61]. This modification can be detected within 15 min after exposing cells to UV irradiation and persists for about 8–12 h [60]. POLR2A ubiquitination requires C-terminal domain phosphorylation, which is a characteristic of elongating POLR2A [61, 62]. Interestingly, the polyadenylation factor CstF associated to the tumor suppressors BRCA1/BARD1 play a role in the proteasome-mediated degradation of POLR2A during DDR [27, 63]. Both the interaction of CstF and BRCA1/BARD1 complex [24, 27, 31] and the proteasome-mediated degradation of POLR2A [27, 63] contribute to the inhibition of 3ʹ end processing that occurs after DNA damage. Those studies suggested the existence of several, possibly redundant, mechanisms to explain the inhibitory effect of UV irradiation on mRNA 3ʹ processing. The studies presented here add another level of complexity indicating that the UV-induced decrease in POLR2A protein levels might be due to intronic APA, which results in a decrease in full-length POLR2A mRNA, supporting the idea that U1 snRNA plays a role in this response. The cellular function of this shorter form of POLR2A protein in cellular transcription as well as other factors involved in intronic APA during DDR will be addressed in future studies. Notably, it has been shown that UV damage switches the usage of pAs from the proximal to the distal one in the 3ʹUTR for the yeast largest subunit of POLR2A gene [64]. How APA-medicated regulatory mechanisms vary in different species is another subject of future investigation.

Although it is known that a differential regulation of genes involved in DDR occurs during the progression of DDR, the mechanisms driving this differential regulation are not completely understood. One example is the p53 pathway. Although p53 binds to the promoter of all of its target genes not all these genes are activated with same stimulus at the same time of the response [65–67]. For example, while UV treatment induces p53 binding at the CDKN1A promoter, it does not induce a strong increase in CDKN1A levels of full-length mRNA and protein [67]. However, p53 can activate CDKN1A expression under cell cycle arrest conditions induced by doxorubicin or Nutlin-3 treatment, indicating that other mechanisms contribute to the regulation of CDKN1A mRNA levels on UV treatment. Previous studies have shown that this stimulus specific response is regulated by posttranslational modifications of p53 [68, 69], p53-binding factors [70, 71], chromatin structure and transcription factors [61]. This suggests that multiple mechanisms contribute to define p53-dependent transcriptional profiles. Our studies indicate that activation of intron-APA and accumulation of shorter mRNA isoforms of CDKN1A might provide an alternative explanation of CDKN1A regulation during UVC-induced DNA damage.

Together, our study reveals a significant gene-specific regulatory scheme in DDR where U1 snRNA impacts gene expression via APA.

## Materials and Methods

### Tissue culture methods

RKO, RKO-E6 and HCT116 cell lines were cultured in Eagle's minimal essential medium and Dulbecco’s modified Eagles medium, respectively. Media were supplemented with 10% fetal bovine serum and 1% penicillin/streptomycin antibiotic.

### DNA-damaging agents

Ninety percent confluent cultures were exposed to UV and harvested at the indicated times. UVC doses (40 J m^−^^2^) were delivered in two pulses using a Stratalinker (Stratagene, La Jolla, CA, USA). Prior to pulsing, medium was removed and replaced immediately after treatment.

### 3ʹREADS

Colon carcinoma RKO cells were treated with UV irradiation and allowed to recover for 0.5 or 2 h. Nuclear RNA was purified using the RNeasy kit (Qiagen, Valencia, CA, USA) following manufacturer’s protocol, followed by 3ʹREADS analysis as described in ref. 33. Briefly, after RNA fragmentation, poly(A)-containing RNA fragments were captured onto magnetic beads coated with a chimeric oligonucleotide (oligo CU_5_T_45_), which contained 45 thymidines (Ts) at the 5′ portion and 5 uridines (Us) at the 3′ portion, and were released from the beads by RNase H treatment, which also eliminated most of the As of the poly(A) tail. Eluted RNA was ligated to 5′ and 3′ adapters, followed by reverse transcription, PCR amplification and deep sequencing.

### Data analysis

Reads from 3ʹREADS were aligned to the genome using Bowtie2 [72], and those with at least two non-genomic As at the 3′ end were considered as PolyA site-supporting reads, which were used for APA analysis. We used the Fisher’s exact test to compare isoform expression between two samples. For 3ʹUTR-APA analysis, the two most abundant 3ʹUTR pA isoforms were compared; for intron-APA analysis, we either compared each intronic pA isoform with all isoforms using pAs in the 3ʹ-most exon or combined all intronic pA isoforms and compared them with all isoforms using pAs in the 3ʹ-most exon. We used *P*-value<0.05 and relative abundance change>5% to select significantly regulated APA events. GO analysis (http://www.geneontology.org) were carried out using Fisher’s exact test. IPA analysis was carried out using default values (www.qiagen.com/ingenuity). Features of introns with regulated pAs were based on RefSeq annotations. 5ʹSS and 3ʹSS scores were based on the maximum entropy method [73].

### Analysis of endogenous mRNAs or APA isoforms abundance

Nuclear RNA was purified from different cell lines using the RNeasy Mini Kit (Qiagen) according to the manufacturer’s directions. RNA concentrations of the samples obtained under different conditions were equalized. Equivalent amounts of purified RNA (2 μg) were used as a template to synthesize cDNA using either random hexamer primers or oligo-d(T) primers and GoScript reverse transcriptase (Promega, Madison, WI, USA) according to the manufacturer’s protocol. PCR was performed using the reverse transcriptase products and Taqman master mix (Applied Biosystems, Foster City, CA, USA). The primers used to detect intron/full-length and distal/proximal mRNA isoforms are described in Supplementary Table S3. Commercially available primers for GAPDH (Applied Biosystems) were used in the qRT–PCR reactions to normalize qRT–PCR reactions. SYBR green master mix (Applied Biosystems) was used in the qRT–PCR reactions. Relative levels were calculated using ΔCτ method. Genomic DNA was prepared from HCT116 cells as in ref. 74. For detection of second introns the following primers were used: POLR2A: forward primer 5ʹ- GGGAAGCAGGCTGGAATTGG-3ʹ, reverse primer 5ʹ- GTCTGCATTGTACGGAGTT-GTC-3ʹ. CDKN1A: forward primer 5ʹ- GAGTGGACGTTCCCCGAGTT-3ʹ, reverse primer 5ʹ- GTCAGCCAGGCCAAGAAGAAG-3ʹ. Ephrin B2: forward primer 5ʹ- GCCAGGAAGGAGGTATAATTGGG-3ʹ, reverse primer: 5ʹ- ACCTTTCTTCTCCCCTGCTAC-3ʹ.

### Depletion and overexpression of U1 snRNA

HCT116 cells were transfected with either 10 nmol of control oligo or U1 snRNA targeting morpholino oligonucleotides according to the manufacturer’s instructions (Gene Tools, Philomath, OR, USA) using scrape delivery method. After addition of morpholino nucleotides, cells were scraped and transferred to a new six-well plate. The plasmid overexpressing U1 snRNA was kindly provided by Dr Gunderson (Rutgers University). HCT116 cells were transfected with this plasmid using Lipofectamine 2000 (Invitrogen, Carlsbad, CA, USA) according to the manufacturer’s instructions. Cells were harvested 48 h after transfection and nuclear RNA was isolated. cDNA was prepared and used in qRT–PCR reactions as described above.

### Nuclear extract preparation

After UV treatment, nuclear extracts were prepared from harvested cells essentially as described [30, 32].

### Cell death ELISA assay

Fragmentation of DNA after induction of apoptosis was determined by photometric enzyme immunoassay (Cell Death Detection ELISAPLUS; Roche, Indianapolis, IN, USA) as recommended by the manufacturer.

### Availability of supporting data

The data sets supporting the results of this article are available in the NCBI’s Sequence Read Archive: http://www.ncbi.nlm.nih.gov/geo/query/acc.cgi?acc=GSE71801.
