# Supplementary material for: Intronic cleavage and polyadenylation regulates gene expression during DNA damage response through U1 snRNA
Source: Cell Discov. 2016 Jun 14;2:16013–. doi: 10.1038/celldisc.2016.13 (PMC4906801; doi:10.1038/celldisc.2016.13)
Supplement: Supplementary information [file celldisc201613-s1.pdf]

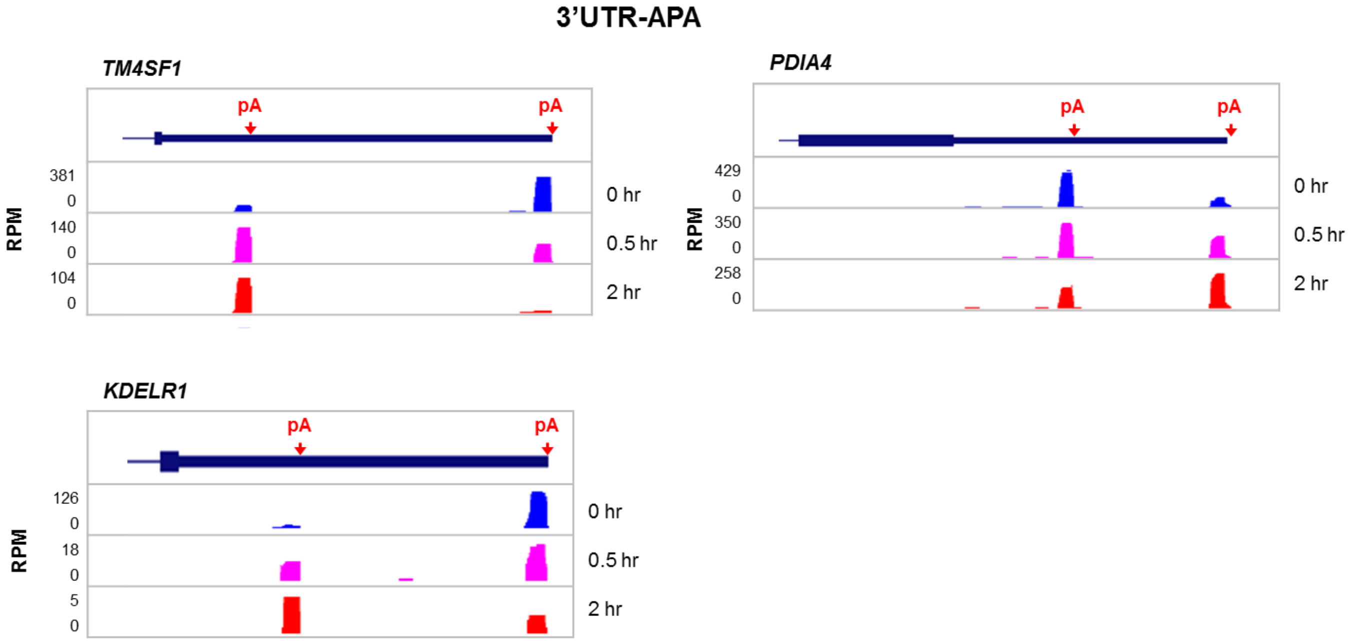

Supplementary information, Figure S1. Examples of genes with regulated 3'UTR-APA in RKO cells. Examples of 3'UTR-APA regulation. The zoomed-in 3'-most exon are shown on the top. pAs are indicated, and their RPM values at different time points are shown.

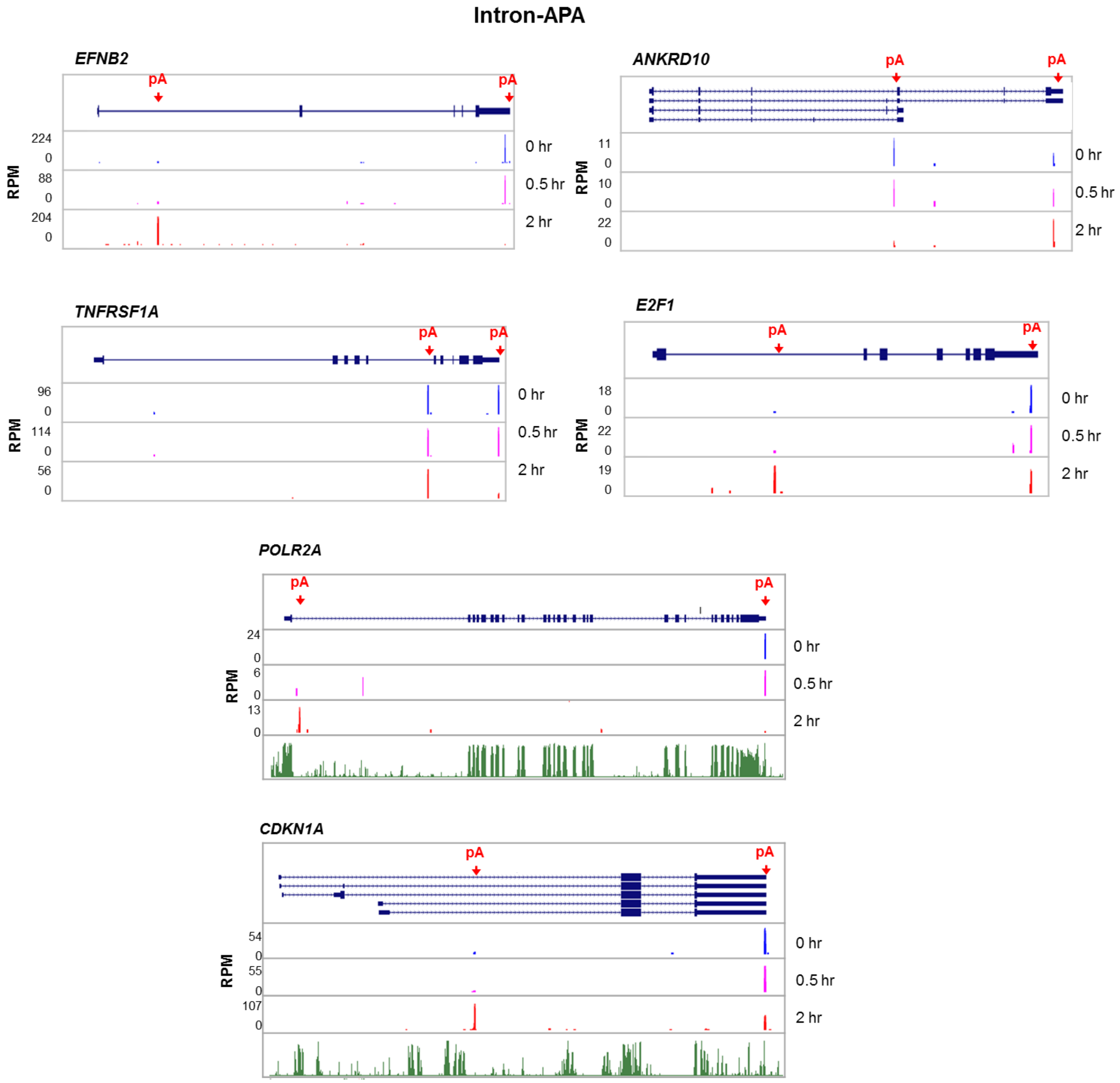

Supplementary information, Figure S2 Examples of genes with regulated intronic-APA in RKO cells. As in Figure S1 except that regulated intronic pAs are shown along with the major pA in 3'UTR. For *POLR2A* and *CDKN1A*, sequence conservation of the shown region (based on mammals) is indicated, with the height of line reflecting the degree of conservation.

A

	Associated Network Functions (IPA)	score
1	DNA Replication, Recombination, and Repair, Cell Cycle, Cellular Growth and Proliferation	31
2	Cancer, Hematological System Development and Function, Organismal Functions	29
3	Protein Synthesis, Cell-To-Cell Signaling and Interaction, Cellular Growth and Proliferation	28
4	Gene Expression, Cell Cycle, Cellular Development	28
5	Neurological Disease, Protein Degradation, Protein Synthesis	28

B

DNA Replication, Recombination, and Repair, Cell Cycle, Cellular Growth and Proliferation

Gene Expression, Cell Cycle, Cellular Development

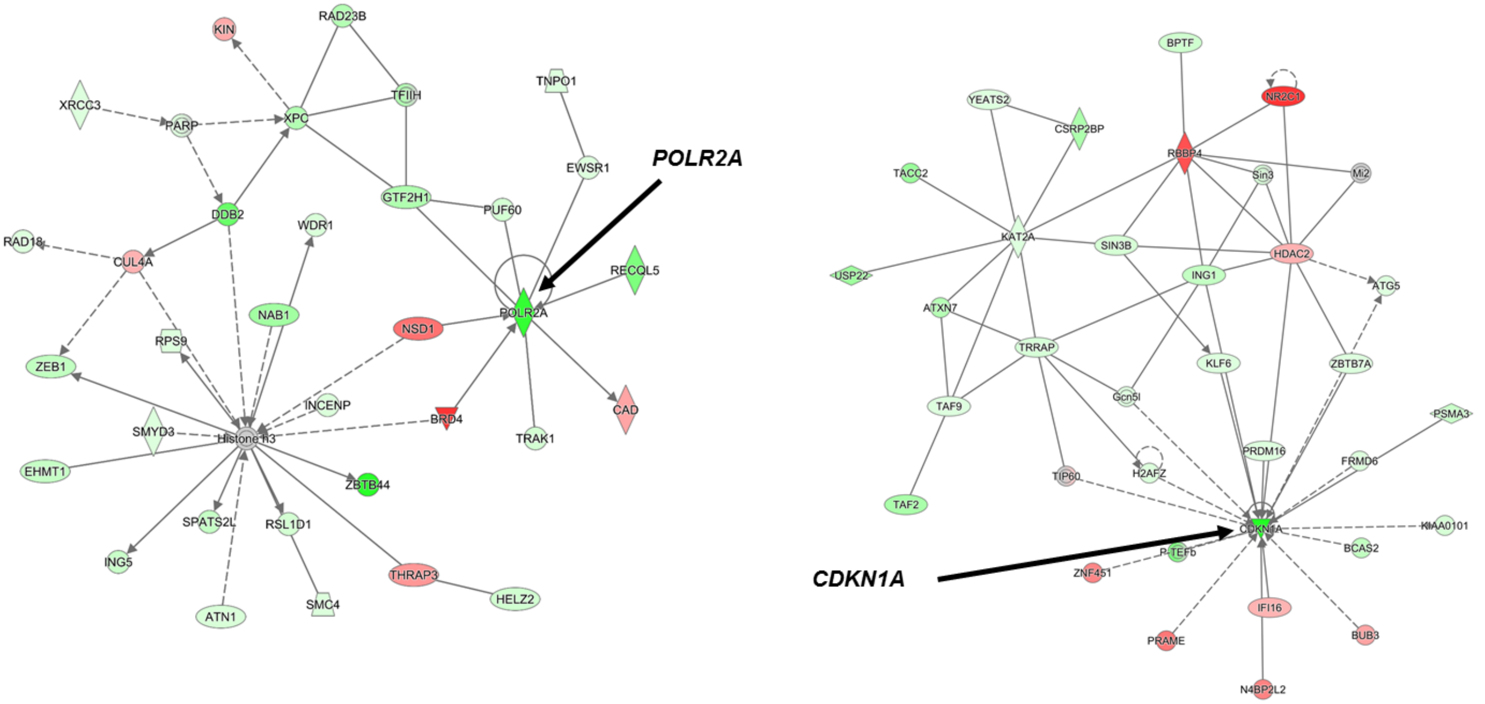

Supplementary information, Figure S3 IPA network analysis of genes with upregulated intronic pA isoforms in RKO cells (A) Top 5 significant gene networks. **(B)** POLR2A (RNAP II) and CDKN1A (p21) are in the two significant networks. The color intensity of a node indicates the degree of its regulation. Red indicates downregulation of intronic pA isoform, green upregulation of intronic pA isoform, and gray no regulation. Dashed line indicates direct interaction of two genes, and dotted line indicates indirect interaction of two genes.

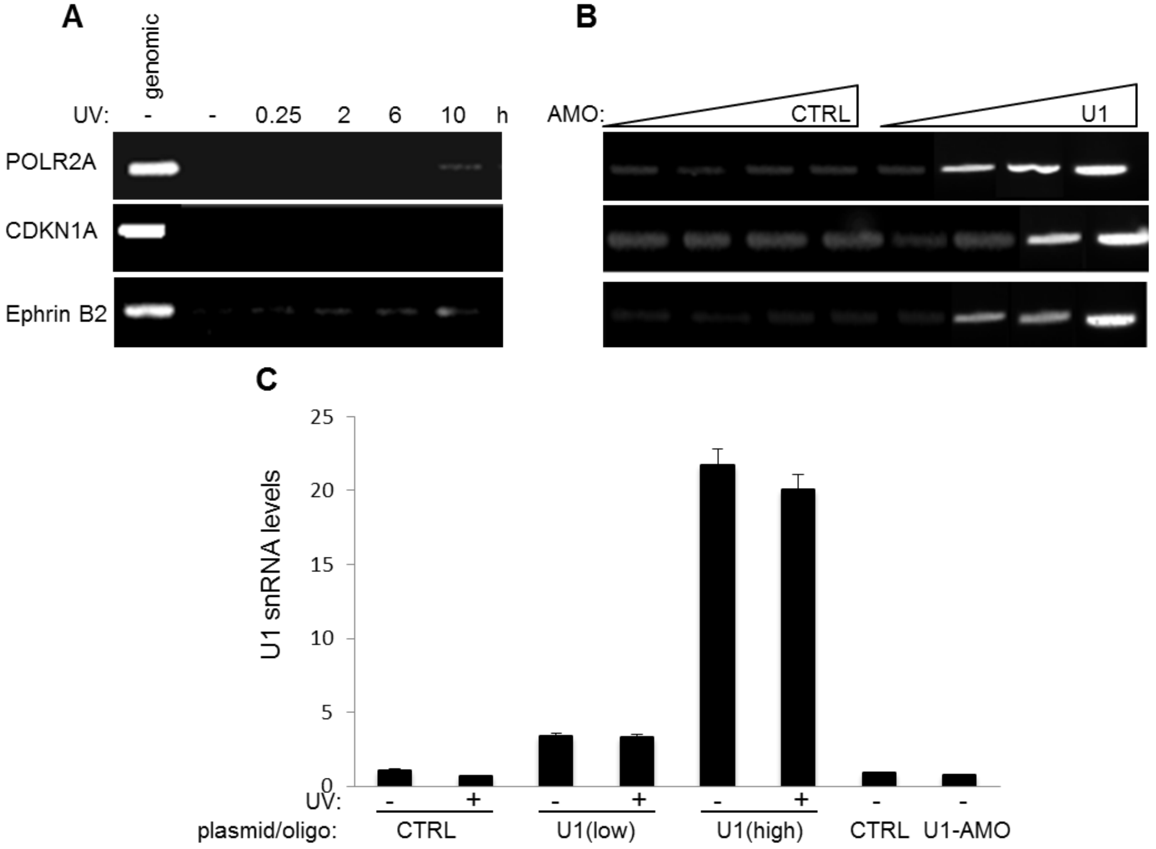

Supplementary information, Figure S4. A) Second introns were not present in the mRNA isoforms from POLR2A, CDKN1A and Ephrin B2 isolated from UV treated cells. HCT116 cells were treated with UV irradiation and allowed to recover for indicated times, and then harvested. cDNAs were prepared using oligo(dT) primers from nuclear RNA and used for PCR reaction with primers specific for second intron. Genomic DNA was also analyzed as control of the PCR reaction. A representative gel from three independent samples is shown. **B) Second introns were detected in the mRNA isoforms from POLR2A, CDKN1A and Ephrin B2 isolated from cells treated with different concentrations of U1 AMO.** Cell were treated with 10, 15, 20 or 25 nmoles of control oligo or U1 snRNA targeting morpholino oligonucleotides using scrape delivery method. **C) Determination of U1 snRNA levels decrease in different cellular conditions.** Samples from HCT116 cells transfected with 10 nmoles of control or anti-sense morpholino targeting U1 snRNA (U1 AMO) and UV treated were analyzed.

Samples HCT116 cells transfected with two concentrations (0.02 μg or 1 μg) of either control or U1 snRNA expressing vectors, and UV treated, were also analyzed. Nuclear RNA was isolated and cDNA was prepared using random primers. qRT-PCR reaction was performed with primers specific for U1 snRNA. qRT-PCR products of actin were used as endogenous control. The qRT-PCR values were calculated from three biological samples by triplicate in each determination.

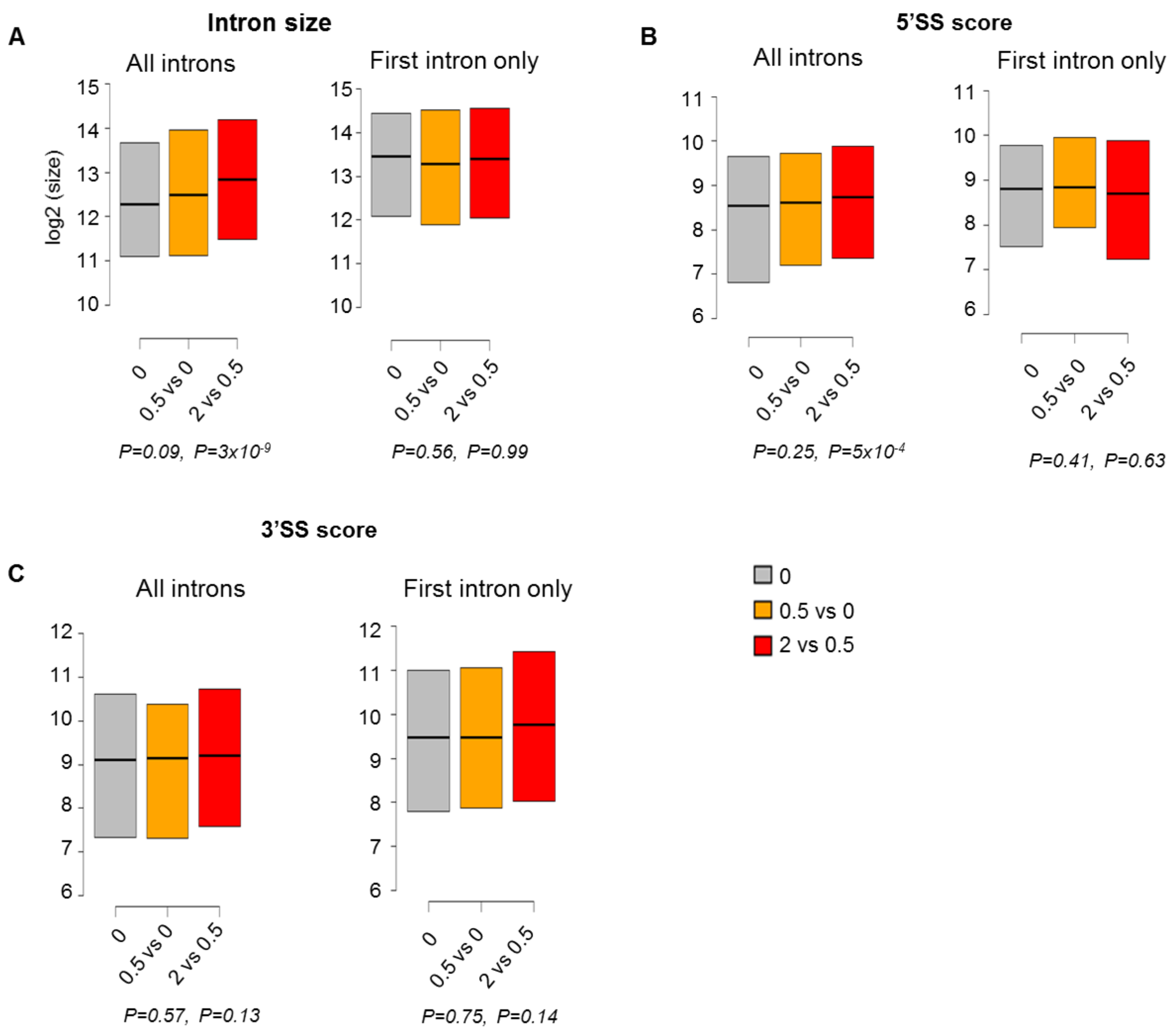

Supplementary information, Figure S5. Features of introns containing pAs. (A) Comparison of intron size. All introns containing pAs at 0 hr are used as control. Introns containing activated pAs are used for the 0.5 vs. 0 hr and 2 vs. 0.5 hr groups. All introns (left) or first introns only (right) are compared. P-values are derived from Wilcoxon test comparing with the control group (0 h) and two other groups. Single introns are excluded for this analysis. **(B)** Comparison of 5'splice site (5'SS) score. **(C)** Comparison of 3'SS score.

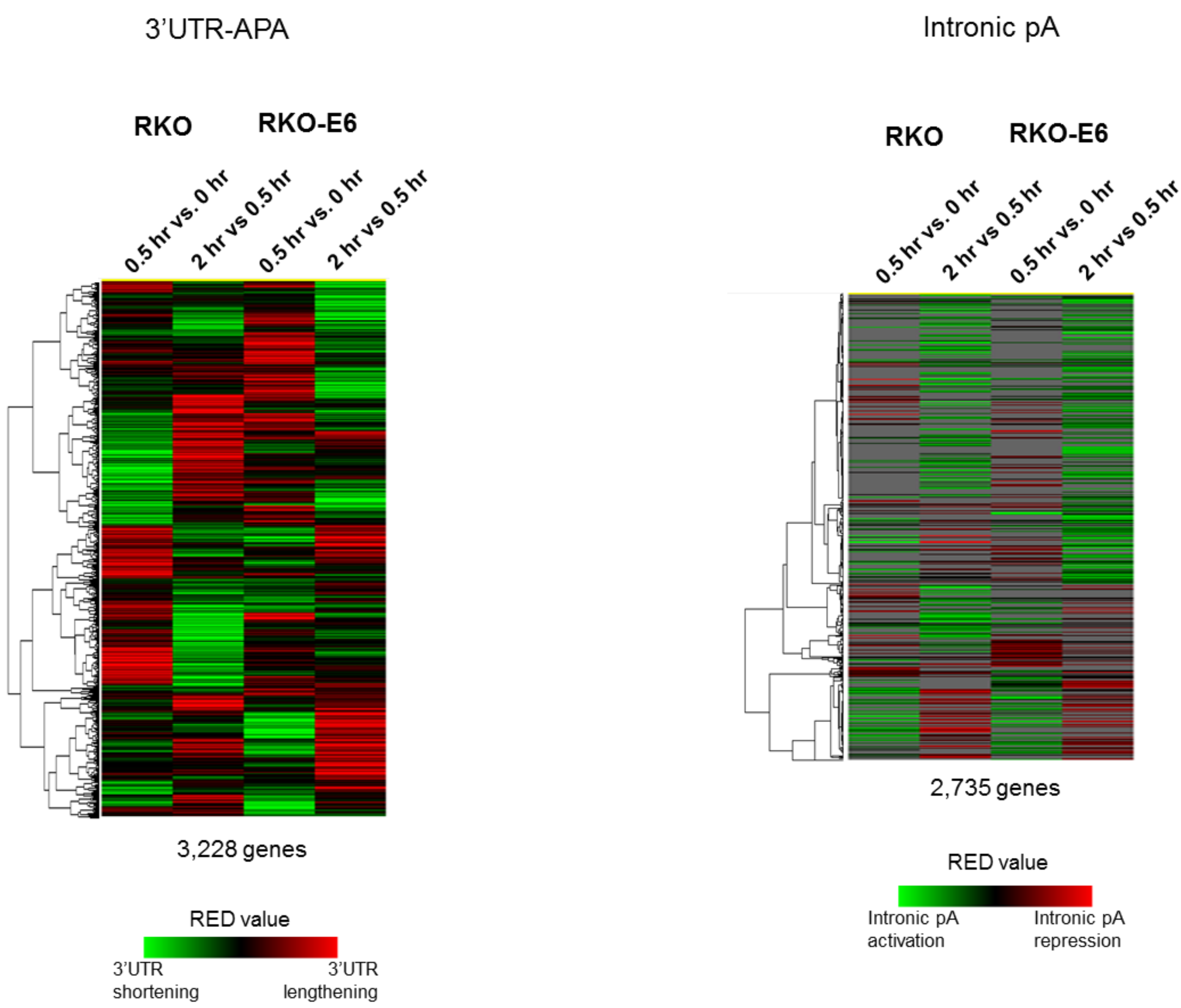

Supplementary information, Figure S6. Heatmaps showing APA regulation in RKO and RKO-E6 cells. Red and blue colors indicate upregulation and downregulation of distal pA compared to proximal pA. Gray color indicates 'NA' value which derives from no expression in one sample. Genes were clustered by hierarchical method using Pearson correlation coefficients. 3'UTR-APA (left panel) and intron-APA (right panel).

Table 1. GO terms associated with genes with regulated 3'UTR-APA

GO ID	GO name	-log$_{10}$(*P*)	
		**0.5 vs. 0 hr**	**2.0 vs. 0.5 hr**
GO:0045454	cell redox homeostasis	0.6	5.9
GO:0070972	protein localization to endoplasmic reticulum	3.4	1.9
GO:0019725	cellular homeostasis	1.0	3.2
GO:0051051	negative regulation of transport	3.2	0.1
GO:0007565	female pregnancy	3.1	0.7
GO:0033619	membrane protein proteolysis	3.0	0.8
GO:0051128	regulation of cellular component organization	0.6	3.0
GO:0009141	nucleoside triphosphate metabolic process	0.4	2.9
GO:0051588	regulation of neurotransmitter transport	1.3	2.8
GO:0043408	regulation of MAPK cascade	2.7	1.4

Supplementary information, Table 1. Top GO terms associated with genes with regulated 3'UTR-APA.

Table 2. GO terms associated with genes with regulated intronic-APA

GO ID	GO name	$-\log_{10}(P)$	
		0.5 vs. 0 hr	2.0 vs. 0.5 hr
GO:0006357	regulation of transcription from RNA polymerase II promoter	0.1	5.3
GO:0006974	response to DNA damage stimulus	0.0	4.0
GO:0021761	limbic system development	2.3	0.1
GO:0006913	nucleocytoplasmic transport	0.5	3.6
GO:0045665	negative regulation of neuron differentiation	0.2	3.5
GO:0051896	regulation of protein kinase B signaling cascade	3.3	0.3
GO:0051817	modification of morphology or physiology of other organism involved in symbiotic interaction	3.3	1.3
GO:0070588	calcium ion transmembrane transport	0.0	3.3
GO:0006997	nucleus organization	0.2	3.2
GO:0042176	regulation of protein catabolic process	0.3	3.0

Supplementary information, Table 2. Top GO terms associated with genes with regulated intronic-APA.

Table 3. Primers used in PCR reactions shown in Figure 2.

Gene	Forward primer	Reverse primer
POLR2A	5'-TGCGCACCATCAAGAGAGTCCA-3'	intron-APA: 5-CCTCCTTCTCACCCTCCAGCCA-3',<br>FL mRNA: 5'-GCGGCCAGTCCGCTCAATCA-3'.
CDKN1A	intron-APA: 5'-GGCGGAGAGCGGGATTACAAGT-3'<br>FL mRNA: 5'-TGAGAGGTTCCTAAGAGTGCTGGGC-3'	intron-APA: 5'-AGGTGGTGGACACAGTGGCGTA-3'<br>FL mRNA: 5'-TGACAGCGATGGGAAGGAGCCA-3'
Ephrin B2	5'-CGTGTGGAAGTACTGCTGGGGT-3'	intron-APA: 5'-AAAGGCGGAGAGACCGTCCGTT-3', FL mRNA: 5'-CAGTTTTAGAGTCCACTTTGGGGCA-3'.
E2F1	5'- TGACCTGCTGCTCTTCGCCACA-3'	Intron-APA: 5'-CTGGCTTGAAGTCGCCCAAAG-3'<br>FL mRNA: 5'-GTCAGTTTCCAGGTCCAGCCT-3'
DSCR3	5'-GGACCGCCCTGGACATCAAGAT-3'	Intron-APA: 5'-TCAAGGGGCCAGATGGGAAGAG-3'<br>FL mRNA: 5'-GTTACCCTTCAAGTGCAGAGG-3'
Notch1	5'-AACGTCTCCGACTGGTCCGA-3'	Proximal: 5'-TGGCATCCACAGAGCGCACACAGA-3'<br>Distal: 5'-GAAGGTGAGCCAGCTTTGCCT-3'
KDELR1	5'-GGAAGGCGGCAGAAGATGAAGAG-3'	Proximal: 5'-CAAGACCTGGATCCTCCACTG-3'<br>Distal: 5'-GATGGGTGTCGGCAGATTTAGTG-3'
DUSP6	5'-ACACCAAATCATGGGCTCACTT-3'	Proximal: 5'-GAGGTGACACTCCCTGAAGAAT-3'<br>Distal: 5'-CCTTGCCCTACTATGCCTACAA-3'
SNRPB2	5'-TAATCAGTTCCCTGGCTTCAAGG-3'	Proximal: 5'-CACCAAGGCTAAAGAAACACTGG-3'<br>Distal: 5'-GATGATAGGGAGATGGGTCAATC-3'

Supplementary information, Table 3. Primers used in PCR reactions shown in Figure 2.
